# Gaze cues (repeatedly) fail to influence person evaluation

**DOI:** 10.1177/17470218251333425

**Published:** 2025-03-28

**Authors:** Samantha E A Gregory, Vilma Pullinen, Margaret C Jackson

**Affiliations:** 1 School of Health and Society, University of Salford, Salford, UK; 2 Department of Psychology, University of Aberdeen, Aberdeen, UK

**Keywords:** Eye gaze, joint attention, person evaluation, social value, ostracism, gaze cuing

## Abstract

Eye gaze is an important social signal that people generally cannot help but follow, leading to joint attention. Joint attention has been shown to speed basic processing of objects, enhance memory for them, and even affect immediate value-based appraisal by increasing object likability. Here, across 8 experiments, we investigate for the first time whether jointly attending to other faces positively affects their longer-term social value (liking, trust) and attentional value (attention allocation and prioritisation). Emanating the basic gaze cuing paradigm, a central cue face looked towards or away from a ‘target’ face, which the participant had to respond to. Unbeknownst to participants, some target faces were always looked at (jointly attended – high value) and others were never looked at (‘ignored’ – low value). In studies 1 to 6, we investigated how these gaze-induced value conditions positively affected subsequent liking and trust social judgements of a person. Then, in studies 7 and 8, we additionally investigated whether effects of gaze on others may occur implicitly, affecting subsequent attentional engagement with others by using the target faces as gaze cues, or attentional targets in a dot probe task. Confirmed through mini meta-analysis, we found no significant effect of being jointly attended versus ignored on either the social (*N* = 214) or attentional (*N* = 77) value of faces. We discuss whether faces are different from objects in this context.

Eye gaze is an important social cue that signals what another person is interested in and allows us to make assumptions about what a person might do next (Land & Tatler, 2009). Extensive research demonstrates that people will often follow the eye gaze of others, thus engaging in joint attention (Frischen et al., 2007), and mentalising where we make inferences about others’ intentions (Capozzi & Ristic, 2018). Primates use eye gaze to signal social hierarchy, with more dominant animals being looked at more often than the lower status members of the group (Chance, 1967; McNelis & Boatright-Horowitz, 1998). Further, primates use gaze aversion to reject interaction and mutual gaze to facilitate it (Chance, 1967; Johnson & Karin-D’Arcy, 2007). Indeed, eye gaze is a complex social signifier in humans where direct gaze can signal attentiveness between two individuals (Freeth et al., 2013), but can also signal dominance (Strongman & Champness, 1968).

Researchers have studied the effects of communicative gaze using a Posner style (Posner, 1980) gaze cuing paradigm whereby a participant views an on-screen face which looks to the left or right before a target item is shown in the looked-at (valid) or looked-away-from (invalid) location. Findings demonstrate that the gaze cue changes the way that cued items are processed, with items in the looked-at location being responded to, and thus processed, faster, even though the cue is uninformative (Frischen et al., 2007). Further, gaze-cued items are found to be remembered better in both working memory (Gregory et al., 2022; Gregory & Jackson, 2017, 2019) and long-term memory (Dodd et al., 2012). In addition, gaze-cued objects also appear to be liked more than objects looked away from by the gaze cue (Bayliss et al., 2006, 2007; Capozzi et al., 2015; Manera et al., 2014).

Unlike the influence of gaze on attention orienting, the effect of gaze on likeability judgements has only been found for gaze cues, and not arrows, indicating that the effect is social in nature (Bayliss et al., 2006). In studies investigating this effect, participants classify common household objects as kitchen or garage items, which are looked at or away from by a central face multiple times before, in a final block, also rating the likability of items. Unbeknownst to participants, the rated target items were always looked at by the cue face (jointly attended), or always looked away from (‘ignored’) during the initial categorisation task. Results show that looked-at items are rated more favourably than looked-away-from items. This effect of gaze on object likability has been found to be abolished by barriers obstructing the face’s view of the items, thus requiring the face to have ‘seen’ the items, indicating that the effect relies on perspective taking (Manera et al., 2014). The effect is also influenced by the trustworthiness of the face (King et al., 2011) and by how many faces are present (Capozzi et al., 2015). In addition, research using food items as targets has demonstrated that people are willing to pay more for food (Madipakkam et al., 2019) and show a stronger desire for water brands (Van Der Weiden et al., 2010) shown under joint attention. This research indicates that in sharing the focus of someone’s gaze, value-based information is conferred to the items of interest.

The effect of joint attention on object value has led to the question of whether gaze cuing can also affect the judgement of people. Indeed, is it possible that if we see someone regularly attended to by other people, we will consider them to be of higher social value than someone who is frequently ignored? Park and Park (2015) have demonstrated using a cyberball task that viewing social exclusion leads to the victim of the exclusion being seen as less human and having lower mental capacity compared to perpetrators. However, research into the effect of eye gaze on judgements of others has shown mixed results. For example, in a study where individuals were presented as part of a natural scene (rather than a gaze cuing paradigm), it was found that individuals looked at by another were rated as more trustworthy than the person looking at them (Kaisler & Leder, 2016). However, here they did not directly compare against a looked-away-from condition and, in a later follow-up where the target individuals were gazed at or away from, there was no effect of gaze on person judgement found (Kaisler et al., 2020). Gaze has, however, been found to affect attractiveness judgements. Jones et al. (2007) had participants rate pairs of images of men before and after showing images of a woman looking at one of the men from each pair, while looking away from the other, with a smiling or neutral expression. Following this, participants again rated the attractiveness of the pairs of faces. Female participants rated the men looked at by the smiling woman as most attractive, reportedly showing mate choice copying effects, while male participants rated those looked at by the smiling woman as less attractive, reportedly showing within sex competition. This, therefore, indicates that certain social cues can affect how others are physically perceived.

However, research using a paradigm that more closely matched the Bayliss et al (2006) object desirability study showed no effect of gaze condition (jointly attended vs. ignored faces) on person ratings but did replicate the effect of gaze on object liking (Landes et al., 2016). Here, participants viewed a central expressive (positive or negative) cue face look either at or away from a neutral target face. Participants then either categorised a character that was presented over the target face as an ‘x’ or a ‘c’ (experiment 1) or categorised the target faces as male or female (experiment 4). They did this for two blocks, followed by a categorisation block where, just after the cuing process and target task, they also rated the likeability of target faces on a scale of 1 (didn’t like at all) to 9 (like very much). While they found a basic gaze cuing effect wherein the target task was conducted more quickly when cue was valid versus invalid, face liking ratings did not show modulation by gaze cuing condition. Landes et al. (2016) used a shorter 250 ms stimulus onset asynchrony (SOA) between the initial gaze cue and the appearance of the target face, compared to 500 ms SOA used in Bayliss et al. (2006), which could explain why no effect of joint attention on face evaluation was found, as research suggests that gaze cuing is strongest between 300 and 750 ms SOA (Frischen et al., 2007). However, importantly, they did find an effect of shared gaze on object liking using these parameters. It is therefore still unclear how and when engaging in socially motivated joint attention to include or ignore another may affect that person’s social value.

It is also important to think beyond how joint attention may confer or infer the social value of others (e.g. in the form of attractiveness or likeability) to consider the impact on ‘attentional value’, for example, relating to perceptual salience and attentional processing resource requirements. Shteynberg (2010) describes a ‘social tuning’ effect where information jointly processed or experienced as part of a social group is more prominent both cognitively and behaviourally, reflected in enhanced processing speed and recognition of words and paintings. In the current study, in addition to considering whether jointly attended faces would be imbued with ‘social value’ in the form of judgements of liking and trust, we also consider for the first time whether jointly attended faces would be imbued with greater ‘cognitive value’ than ignored faces, reflected in the inherent ability of those faces to subsequently draw and guide spatial attention.

One important distinction of our approach that diverges from previous work is that rather than ask participants to evaluate faces immediately after the cue, we wanted to examine whether repeatedly seeing someone being looked at versus ignored by another builds up longer-term value-based learning effects. For example, findings that jointly attended objects are liked more when judgements are made directly after the cuing process (e.g. Bayliss et al., 2006; Landes et al., 2016), tell us that in that attentional window and moment they are perceived as more likeable. This does not, however, evidence whether the inherent value of that object has been fundamentally changed to affect behaviour or judgement should that object be re-encountered. Indeed, it may be that an effect would have been seen in Landes et al. (2016) if faces had been rated separately from the cuing stage. It is important to consider whether person impressions can be modulated in the longer term by joint attention or perceived ostracism, as this can have significant consequences for how we may choose to interact (or not) with others both during the current social interaction and in future interactions with the same individuals. So, as per Madipakkam et al. (2019), where ratings of willingness to pay for food were conducted in isolation from the gaze cuing process, we investigated if the effects of gaze are transferred to the person when examined in a separate social or cognitive value measurement phase.

Across 8 experiments, we investigated the effect of jointly attending versus ignoring other faces on their subsequent social value (judgements on liking, trust, and competence; experiments 1–6) and cognitive value (measured via attentional bias and gaze cuing ability; experiments 7–8). In all experiments except experiment 6, in Phase 1 (called the ‘value learning’ phase), participants judged the age of target faces that a central cue face either always looked towards (jointly attended – what we call ‘high value’ faces throughout) or always looked away from (ignored – what we call ‘low value’ faces throughout). Participants were not informed about this value manipulation. Social value (person judgements) and cognitive value (attentional properties) of the target faces were measured in Phase 2 in a separate block just after Phase 1 ended. In experiments 1 to 5, we conducted a variety of cue and target face manipulations in Phase 1 to explore the conditions under which social value may be modulated by shared gaze. Experiment 1 used a traditional subtle eye gaze shift. Experiment 2 used a more obvious head turn. In experiment 3, cue faces were expressive (happy and disgust). In experiment 4, the target faces made a ‘bid for attention’ by looking towards the central cue face before having their bid either returned (high value – they were looked at by the cue face) or rejected (low value – the cue face turned away from them). In experiment 5, the target faces always first looked away from the central cue face, then either had their gaze followed by the cue (high value) or not (low value – cue face looked in the opposite direction and thus away from the target face). In experiment 6, we measured liking for faces immediately after the gaze cuing sequence used as per experiment 2, thus swapping the age rating for immediate value rating on a trial-by-trial basis, rather than in a separate phase, reflecting the task used by Bayliss et al. (2006).

In experiments 7 and 8, we investigated the effect of gaze cues on cognitive value by measuring the faces’ subsequent ability to affect attention. Here, we used head turn gaze shifts and a neutral expression in Phase 1. In experiment 7, in Phase 2, the target faces that were either looked at or looked away from in Phase 1 became the gaze cues in a traditional target localisation cuing task; in experiment 8, the target faces became probes in a standard dot probe task. We expected in experiment 7 for target faces that were always looked at in Phase 1 to provide more powerful gaze cues themselves in Phase 2, indexed by increased gaze cuing effect magnitude in comparison to faces that were never looked at. In experiment 8, we expected that faces always looked at (high value) would be preferentially attended to in Phase 2 when paired with faces that were always ignored (low value), indexed by faster reaction times to locate a simple probe (two dots) that inhabited the previous location of the higher value face. Across all these experiments, we found no evidence for effects of gaze-induced value on social judgement or attention orienting ability of the faces. We present the results of each experiment individually and provide a mini meta-analysis (Goh et al., 2016) of all experiments.

## Experiment 1: person evaluation – gaze shift

In experiment 1, we used the parameters of the traditional gaze cuing paradigm, presenting a central cuing face which looked left or right and, after a 500 ms SOA, showing the target face in either a looked-at (high value) or looked-away-from (low value) location. We also included an uncued condition on a third of trials where the cue face did not change gaze direction; this was considered useful to explore whether any effects of the high/low value manipulation served to increase perceived value or to devalue social judgements relative to the uncued baseline. Participants’ task was to judge the age of target faces. We chose an age judgement task over something like the categorisation task used by Bayliss et al. (2006) to try to encourage participants to properly look at the faces of these individuals. Further, we ensured all faces remained on screen for an equivalent amount of time, to allow for some faces to be responded to more quickly than others. This was to ensure that any effect of gaze on social value would be related to the value condition and not exposure time, as mere exposure to a stimulus can affect judgement (Zajonc, 2001). These age judgements occurred ten times for each target face (randomised) and each face identity was either always seen in a looked-at, or a looked-away-from context. We then had the participants’ rate the target faces separately from the gaze cue condition to see if any effects of gaze were transferred to the faces in isolation. We required participants to rate faces on likability, trust and competence, thus covering the dimensions of social judgement (Abele et al., 2008). We predicted that ratings would be higher for the jointly attended high value vs. ignored low value faces.

### Method

#### Pre-registration

This experiment, along with experiments 3, 4 and 7 were pre-registered on AsPredicted (experiment 1: https://aspredicted.org/RXS_9WD). Note that all pre-registrations were made on the same day, within a few minutes of each other, as they made up a body of work submitted as part of a grant proposal (not funded). Within this registration, we aimed to test the hypothesis that when faces are jointly attended (high value), they will be rated as more likable and trustworthy than when they are not jointly attended (low value). To test this, we stated that we would initially recruit 30 participants (a within subjects design with 27 participants has 80% power; G* Power version: 3.1.9.7; Faul et al., 2007) to detect a medium effect (*d* ≈ 0.5; e.g. Bayliss et al., 2006) and then use Bayesian analysis (conducted in JASP version 0.16.3.0; Love et al., 2015) to determine if more participants should be tested to resolve the question. With Bayesian analysis, using JASPs inbuilt interpretation tables and focusing on *BF*_+0/inc_ (*BF*_+0_ refers to the directional hypothesis that Measure 1 > Measure 2; Van Doorn et al., 2020), *BF*_inc_ relates to the inclusion Bayes Factor for the ANOVA (Van Den Bergh et al., 2020) results are considered anecdotal evidence that the experimental hypothesis (H1) is true when *BF*_+0/inc_ is between 1 and 3, moderate between 3 and 10 and strong above 10. *BF*_+0/inc_ = 1 indicates that the data lends equal support to H1 and the alternative hypothesis (H0). Moderate support for H0 is indicated when *BF*_+0/inc_ is between 0.33 and 0.10, and strong evidence is indicated when *BF*_+0/inc_ ≤ 0.10. This allows researchers to look at their data and determine if more participants are necessary to come to an adequate conclusion. If the Bayes factor was between 0.33 and 3, we intended to continue data collection until the findings pointed towards more evidence one way than the other for the key hypothesis. We additionally dictated that we would have a sampling stopping point of 60 people, from which point it was considered that further collection would be futile.

In the end due to the grant proposal not being funded, data was collected by students completing their final year projects. This resulted in some key changes from the registration, first, to maximise data collection, more than one student used this experiment as the basis of their final year project, this meant that the stopping rule was not applied. Second, to complete their research goals, these students collected additional measures. These were that as well as liking and trustworthiness, they had participants rate competency. Further they collected questionnaire data from participants using the Empathy Quotient (EQ; Baron-Cohen & Wheelwright, 2004) and the cultural orientation scale (Triandis & Gelfand, 1998), these were not analysed here, but data is available online.

#### Participants

We recruited 61 adult volunteers from the University of Aberdeen (19 men, 42 women, mean age 23 years, *SD* 3.3 years, range 18–32 years). All participants had normal or corrected to normal vision, and ethical approval was obtained from the School of Psychology Ethics Committee at the University of Aberdeen. Stimuli were presented using E-prime software version 2.0 (Schneider et al., 2002) on a Dell LCD monitor (32-bit true colour; resolution 1,280 × 1,024 pixels). This was an opportunity sample recruited by final year project students.

#### Stimuli and apparatus

##### Gaze cue faces

We selected a set of 6 faces (3 male, 3 female) with neutral expression to use as gaze cues from the Radboud Faces Database (Langner et al., 2010). Each face identity had three photographed natural gaze states – eyes looking left, right and direct – and were presented in grayscale to limit variation between the distinct direct and averted gaze images presented. Faces were presented in the centre of the screen and face dimensions were 219 × 303 pixels.

##### Target faces

**Figure 1.**
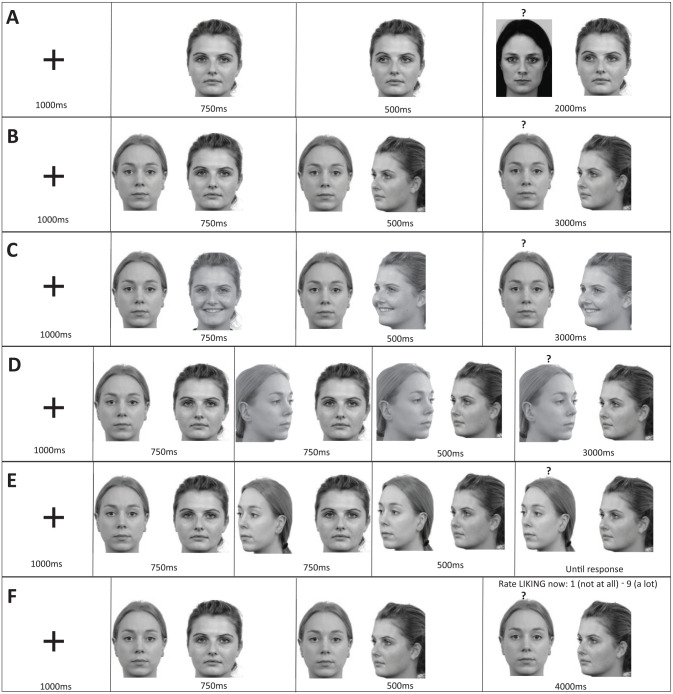
Phase 1: social value learning variations. *Note*. This figure demonstrates the various configurations of the social value learning phase of the experiments (gap between faces is to scale but note cue face always in centre). (A) shows the configuration used in experiment 1, (B) shows the configuration used in experiments 2, 7 and 8, (C) shows the configuration used in experiment 3 (happy condition shown), (D) shows the configuration for experiment 4, (E) shows the configuration for experiment 5, and (F) shows the configuration for experiment 6. All versions are showing the high value condition. Here, the target face is always shown on the left, and note that while only females are shown, face pairs could be mixed gender. (A) uses the face F01NES from the KDEF database (Lundqvist et al., 1998) as the target. For all others the target face shown is Rafd12 and the cue face is Rafd31 from the Radboud database (Langner et al., 2010). Images used in accordance with copyright. KDEF = Karolinska Directed Emotional Faces.

We selected a set of 18 neutral expression faces (9 male, 9 female) to use as targets from the Karolinska Directed Emotional Faces database (KDEF; Goeleven et al., 2008), also presented in grayscale. For all conditions there were equal numbers of male and female faces, 6 faces were high value (always looked at), 6 low value (always looked away from), and 6 neutral (i.e. cue face maintained direct gaze). Target faces were presented 84 pixels to the left or right of the cue faces edge and face dimensions were 219 × 303 pixels. Figure 1 illustrates the trial structure of social value learning in Phase 1.

#### Design

Within subjects’ independent variables were target value (high – always looked at, low – always looked away from, or uncued). The experiment had 6 target faces per value signal condition with 10 exposures each (randomised, counterbalanced) = 180 trials (60 trials per value condition in Phase 1). In Phase 1, the dependent variable was the speed at which the target faces were rated for age. However, the key dependent variable was the rating of the target faces in the subsequent rating phase.

#### Procedure

##### Phase 1: social value learning

Matching Bayliss et al. (2006), we adopted the parameters of the traditional central cuing paradigm where the cue remains on screen for the entire trial (Driver et al., 1999; Friesen & Kingstone, 1998). A fixation cross was presented at the centre of the screen for 1,000 ms, then replaced by the cue face looking direct for 750 ms. This was then replaced by the cue face with the gaze shifted left or right or, in the uncued condition, still showing direct gaze. After 500 ms, the target face appeared (500 ms SOA). Participants were informed their task was to imagine that they were working in a bar and had to judge if the people they saw to the left or right of the central face were older or younger than 21 using the up (older) and down (younger) arrows on the keyboard. They were explicitly told that the central face was a distractor. On valid trials (1/3), the target appeared on the side towards which the cue had shifted, making these faces high value status; on invalid trials (1/3), the target appeared on the opposite side, making these faces low value status. The remaining third of trials were an uncued condition where the cue face did not change gaze direction. A target was present on all trials. The target and the cue remained on screen for the full 2,000 ms so that all faces were seen for an equal amount of time even when a judgement was made quickly. If the judgement took longer than 2,000 ms, the trial ended, and the participant was told they were too slow. The participants were then given feedback related to their response – looked over 21 – serve, under 21 – check ID. For a pictorial representation of the trial structure (see Figure 1A).

##### Phase 2: person evaluation

After the initial social value learning phase was complete participants were told that they would now be ‘shown some of the faces you saw previously and asked to rate them on three traits: to what degree you think they are (1) likeable, (2) competent, and (3) trustworthy’. Then the 18 target faces from Phase 1 were presented to the participants individually in a random order and rated for competence (To what extent do you think this person is Competent?) then likability (To what extent do you think this person is Likeable?) and finally trustworthiness (To what extent do you think this person is Trustworthy?) all on a scale of 1 to 9 (1 = *low*, 9 = *high*). These questions were asked consecutively for each face such that they would see person A and rate them on each trait before seeing person B, and so on. This was different from the Bayliss et al. (2006) study where the items were classified for five blocks of 72 trials, followed by a final 6th block in which participants rated the item they had just categorised on a scale from 1 to 9. Here the ratings were completed separately from the categorisation. Finally, participants completed the questionnaires.

#### Analysis plan

Results here and in subsequent studies are analysed as follows. The Phase 1 social value learning phase is analysed looking at median^1^ reaction time (RT) differences between the looked-at (validly cued) and looked-away-from (invalidly cued) faces. Median RTs are used to avoid the requirement to adjust for outliers and control for the positively skewed nature of reaction times data (Jensen, 1992; Ratcliff, 1993). Results in Phase 1 are largely inconsequential but can tell us something about how the cues are used; statistical outputs are provided in Table 1 for this and all other experiments in which age judgements were measured. The important value ratings are then assessed using the Phase 2 data, with each judgement rating assessed separately. Here we compare the ratings for the high-value faces to the low-value faces only. As per our pre-registration, the uncued condition was included to understand the nature of any differences in high vs low value that may be found. This condition would enable understanding of whether differences were due to the low value face being devalued, or the high value face being valued, and thus does not form part of the initial analysis. However, data for all studies, including questionnaire data, can be found online with the full raw data set and analysis files: https://osf.io/uzc8p/.

**Table 1.** Phase 1: social value learning results.

Experiment	High value	Low value	Uncued	*t*	*df*	*p*	*d*
Experiment 1	919 (139)	926 (139)	941 (146)	0.603	60	.549	0.077
Experiment 2	792 (228)	860 (263)	868 (257)	2.418	24	.024	0.484
Experiment 3 (Happy)	753 (311)	727 (302)	–	−0.877	29	.388	−0.16
Experiment 3 (Disgust)	730 (320)	779 (329)	–	1.520	29	.139	0.278
Experiment 4	759 (226)	759 (234)	790 (252)	0.005	28	.996	<0.001
Experiment 5	1,177 (554)	1,234 (591)	–	1.338	37	.189	0.217
Experiment 7	821 (282)	856 (306)	952 (276)	1.599	27	.121	0.302
Experiment 8	711 (271)	754 (311)	789 (294)	2.379	48	.021	0.34

*Note*. Table shows the median reaction times (standard deviation in brackets) for the looked-at, looked-away-from and uncued conditions, *t*-test results are shown for the comparison between the high value and low value conditions only. For all experiments, the reaction times data was filtered to remove trials where the participant timed out, no other reaction times conditions were applied, data loss due to timing out was <6% in each experiment.

Results are reported for each experiment using standard null hypothesis significance testing with additional analysis conducted with Bayesian statistics using JASP and retaining the standard settings: *t*-test – a half-Cauchy prior distribution (to account for directional hypothesis) scaled to 0.707; ANONA – r-scale fixed effects = 0.5, random effects = 1 and covariates = 0.354 (Version 0.16.3.0; Love et al., 2015). This means that for experiment 1, we are testing the hypothesis that the high value looked-at faces will be rated more favourably than the low value looked-away-from faces. Therefore, while the frequentist test will use a two-tailed test, the Bayesian analysis will be one-tailed to account for the prediction made (see Keysers et al., 2020; Van Doorn et al., 2020).

### Results

#### Phase 2: person evaluation

**Figure 2.**
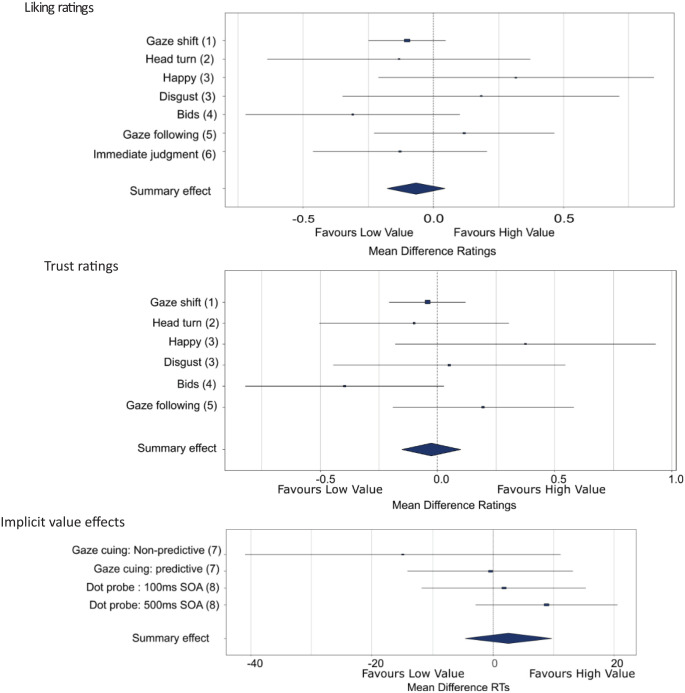
Forest plots showing mean differences (high value compared to low value) for all experiments. *Note*. Figure shows the results summary of mean differences (95% confidence intervals) for the high compared to low value faces in Phase 2 of all experiments (experiment numbers in brackets) for the liking, trust and implicit value effects. For the immediate judgement experiment (6) judgements are combined across the 10 exposure time points. A positive value on the *x* axis favours the high-value faces, and a negative value favours the low-value faces. Plots created using the metaviz library in R (version 4.3.0).

Paired samples *t*-tests comparing ratings between the high value (valid, looked-at faces) and low value (invalid, looked-away-from faces) faces showed no effects of value on any ratings. Liking (high value, *M* = 5.45; low value, *M* = 5.55), *t*(60) = −1.328, *p* = .189, *d* = −0.170 (*BF*_+0_ = 0.064, i.e. *strong support for H0*); trustworthiness (high value, *M* = 5.53; low value, *M* = 5.57), *t*(60) = −0.515, *p* = .608, *d* = −0.066 (*BF*_+0_ = 0.098, i.e. *strong support for H0*); competence (high value, *M* = 5.78; low value, *M* = 5.80), *t*(60) = −0.343, *p* = .0733, *d* = −0.044 (*BF*_+0_ = 0.109, i.e. *moderate support for H0*). Thus, the evidence shows that individuals who are repeatedly looked at are not deemed more competent or liked or trusted more than those repeatedly looked away from. Results for the liking and trust ratings are presented in Figure 2 (competence is not shown as this measure was dropped from all further experiments; its inclusion here is due to the use of final year project students for data collection).

## Experiment 2: person evaluation: head turn

While the results of experiment 1 suggest that eye gaze does not imbue positive social value, the gaze cues used only shifted their eyes to look towards or away from the target face. It is possible that this signal was too subtle to influence the social value of the target faces. Further, averted gaze can have additional social meanings beyond looking at something, for example, averted gaze can signal nervousness (Larsen & Shackelford, 1996), deception (Aavik et al., 2006), or boredom (Kleinke, 1986). It is therefore possible that the cues in the averted gaze condition were not viewed as making a social value statement about the target face, but instead as providing information about how the cue face itself was feeling. Therefore, here in experiment 2, the cue faces make a full head turn to look at the target faces, to improve the clarity of the signal. We again predicted that ratings would be higher for the high value compared to the low-value faces.

### Method

#### Participants and apparatus

Twenty-five adult volunteers were recruited from the University of Aberdeen (7 men, 18 women, mean age 20 years, *SD* 1.63 years, range 18–25 years). This was an opportunity sample recruited by a final-year project student. The use of Bayesian analysis allows us to understand whether the study requires more evidence or is providing evidence already for or against the hypothesis. Stimuli presentation equipment matched experiment 1.

#### Stimuli

##### Gaze cue faces

Again, we selected a set of 6 faces (3 male, 3 female) to use as gaze cues from the Radboud Faces Database (Langner et al., 2010). Here, each face identity had three photographed natural head turned gaze states with their heads looking straight ahead or turned at an angle of 45° from centre to the left or right. In these images, the people are looking frontally from their own perspective (see Figure 1B). Again, these were presented in grayscale, in the centre of the screen, and face dimensions were 219 × 303 pixels.

##### Target faces

To improve visual consistency, we selected an additional set of 18 neutral expression faces (9 male, 9 female) to use as targets from the same Radboud Faces Database (Langner et al., 2010), again presented in grayscale. As with experiment 1, for all conditions, there were equal numbers of male and female faces, 6 faces were high value (always looked at), 6 low value (always looked away from) and 6 neutral (i.e. cue face remained direct). Target faces were presented 84 pixels to the left or right of the cue faces edge, and face dimensions were 219 × 303 pixels.

#### Procedure

##### Phase 1: social value learning

The procedure matched experiment 1, except for the following changes. We presented the gaze cue and target on screen together immediately (unlike in experiment 1 where the cue was presented first, see Figure 1B); this was done to increase realism in the study. After 750 ms, the cues head turned to the left or right, looking at (high value) or away (low value) from the target (or in the uncued condition, remained looking directly). After 500 ms, a question mark appeared above the target to initiate the age judgement. We also increased the exposure time such that participants had 3,000 ms to make the age judgement, again with the faces remaining on screen for the full 3,000 ms regardless of when the response was made, to ensure equal exposure time. Finally, the age rating task was changed from judging if the face was over 21 to judging if they were over 25, as this was felt to better reflect the age range of faces.

##### Phase 2: person evaluation

The procedure matched experiment 1, except here we just had participants rate on trust and liking, removing competence.

### Results

#### Phase 2: person evaluation

As seen in experiment 1, paired samples *t*-tests comparing ratings between the high value (valid, looked at) and low value (invalid, looked away from) faces showed no effects of value on any ratings (see Figure 2). Liking (high value, *M* = 5.01; low value, *M* = 5.14), *t*(24) = −0.514, *p* = .612, *d* = −0.103 (*BF*_+0_ = 0.149, i.e. *moderate support for H0*); trustworthiness (high value, *M* = 5.01; low value, *M* = 5.11), *t*(24) = −0.486, *p* = .631, *d* = −0.097 (*BF*_+0_ = 0.152, i.e. *moderate support for H0*). Thus, we find again that seeing someone repeatedly turned towards or away from does not positively influence subsequent value judgements of that individual.

## Experiment 3: person evaluation: happy vs disgust

While the results of experiments 1 and 2 suggest that there is no effect of eye gaze on person evaluation, the gaze cues used showed neutral facial expressions. Eye gaze without emotional expression is ambiguous (Adams & Kleck, 2005), and this ambiguity may be why there is no effect of gaze on people’s social value. Happiness and disgust are important human emotions, and coupled with eye gaze they can signal important social information. Looking at something with a happy expression indicates that you like the thing you are looking at and are pleased with it, whereas the expression of disgust signals the opposite. When investigating the effect of gaze cues on object liking, Bayliss et al. (2007) found that participants liked objects that were looked at by a cue face with a happy expression more than objects that were looked at with a disgust expression. Further, finding that while items looked at by a happy face were rated more favourably than those looked away from by a happy face, there was no such difference for objects looked at or away from by a disgusted face. They also found that there was no effect of expression on ratings for the objects looked away from by the cue, indicating that value was added by the face looking at the object rather than objects being devalued by the faces looking away. In addition, Jones et al. (2007) examined the effect of eye gaze on attractiveness ratings for pairs of male faces and found that female participants preferred the face of the man that was looked at by a smiling woman compared to the man that was looked away from, with no such effect being found when the woman showed a neutral expression.

It is thus possible that to influence social value, the gaze cues need to be expressive to convey some form of valence judgement onto the target faces. Therefore, here in experiment 3, the cue faces show either a happy or disgusted facial expression and, like in experiment 2, made full head turns. We predicted that there would be an interaction between face expression and value judgement, with those looked at by a happy face being rated more favourably than those looked at by a disgust face. This experiment, including the analysis plan, was pre-registered on AsPredicted: https://aspredicted.org/11M_TGY.

### Method

The method matched experiment 2, except for the following changes. Here the facial expression of the cue showed either happiness or disgust. Further, to keep the design simple and the number of trials manageable, we did not include an uncued gaze condition; therefore, there were 6 target trials per high/low value signal and expression condition repeated 10 times (randomised, counterbalanced) giving 240 trials in total (60 trials per value/expression condition).

#### Participants and apparatus

Thirty adult volunteers were recruited from the University of Aberdeen (9 men, 21 women, mean age 22 years, range 20–27 years). This was an opportunity sample recruited by a final-year project student. No participants were excluded from analyses. Stimuli presentation equipment matched experiment 1.

#### Stimuli

##### Gaze cue faces

Here we selected a set of 12 faces to use as gaze cues from the Radboud Faces Database (Langner et al., 2010). Of the faces, 6 showed a disgusted facial expression (3 male, 3 female) and 6 showed a happy facial expression (3 male, 3 female). As with study 2, each face identity had three photographed natural head turned gaze states with their heads looking straight ahead or turned at an angle of 45° from centre to the left or right. Again, these were presented in grayscale, in the centre of the screen, and face dimensions were 219 × 303 pixels (see Figure 1C).

##### Target faces

The target faces matched the properties seen in study 2.

### Results

#### Phase 2: person evaluation

We conducted separate 2 (happy/disgust) × 2 (high value/low value) ANOVAs for liking and trust ratings, which showed no effects of value or expression (see Figure 2).

Liking: no main effect of expression; *F*(1, 29) = 0.818, *p* = .373, η_p_^2^  = .027 (*BF*_inc_ = 0.163, i.e. *moderate support for H0*), no main effect of social value condition; *F*(1, 29) = 1.139, *p* = .295, η_p_^2^  = .038 (*BF*_inc_ = 0.367, i.e. *anecdotal evidence for H0*) and importantly, no interaction between expression and social value condition; *F*(1, 29) = 0.242, *p* = .627, η_p_^2^  = .008 (*BF*_inc_ = 0.069, i.e. *strong support for H0*).

Trustworthiness: no main effect of expression; *F*(1, 29) = 0.231, *p* = .634, η_p_^2^  = .008 (*BF*_inc_ = 0.149, i.e. *moderate support for H0*), no main effect of social value condition; *F*(1, 29) = 0.844, *p* = .366, η_p_^2^  = .028 (*BF*_inc_ = 0.269, i.e. *moderate support for H0*) and, again, no interaction between expression and social value condition; *F*(1, 29) = 1.433, *p* = .241, η_p_^2^  = .047 (*BF*_inc_ = 0.073, i.e. *strong support for H0*).

Overall, these data provide more evidence for the null hypothesis than the experimental hypothesis. Therefore, our prediction that face expression would affect value judgement was not confirmed.

## Experiment 4: person evaluation. Target faces always ‘Bid for Attention’ first

In experiments 1 to 3, we found that the gaze of another person did not influence the social value of a target person, regardless of whether attention was guided by a subtle eye gaze shift or a more obvious head turn, and regardless of facial expression. This indicates that these social cues alone are not enough to affect the social value of another person. In social interaction, it is often the case that someone is seeking out social interaction through ‘attention bids’ made by looking at another person (Caruana et al., 2020; Freeth et al., 2013). Here in experiment 4, we investigate whether the acceptance or rejection of these bids for attention may influence that person’s social standing, and therefore how much they are liked or trusted by others.

In an attentional bid condition, the target face turns their head to look at the cue face first, and this bid is either reciprocated by the cue face (turns their head to look at the target face) or rejected by the cue face (turns away). If a reciprocated bid for attention serves to raise their social value, then the target will be rated as more likeable and trustworthy than when that bid for attention is actively rejected. In a third condition, the cue face did not respond at all to the attention bid from the target face and remained looking directly ahead (uncued condition). It was predicted that when observed attention bids were reciprocated (attended, high value condition), the bid face would be rated as more likeable and trustworthy than when bids were rejected (ignored, low value condition). This experiment, including the analysis plan, was pre-registered on AsPredicted: https://aspredicted.org/9J5_G8F.

### Method

#### Participants and apparatus

Thirty adult volunteers were recruited from the University of Aberdeen; however, one participant was excluded for failing to successfully complete the task (no useable data in one cue condition); thus, there were 29 participants in the final sample (4 men, 25 women, mean age 22.5 years, *SD* 5.4 years, range 18–42 years). This was an opportunity sample recruited by a final year project student. Stimuli presentation equipment matched experiment 1.

#### Design

Within subjects’ independent variables were social value conditions, (a) High value: bid is reciprocated. (b) Low value: bid is rejected. We also had a third uncued response condition which, as with previous reported experiments, was included for the purpose of testing any high vs low value effects in follow-up and therefore is not analysed here. The experiment had 6 target faces per value signal condition with 10 exposures each (randomised, counterbalanced), resulting in180 trials (60 trials per condition).

#### Stimuli

##### Gaze cue faces

These faces matched the parameters of study 2.

##### Target faces

Here, the 18 target face identities (Radboud Faces Database; Langner et al., 2010) had three photographed natural head turned gaze states with their heads looking straight ahead or turned at an angle of 45° from centre to the left or right (see Figure 1D). For all conditions, there were equal numbers of male and female faces, 6 faces were high value (always had their attention bid returned), 6 low value (always had their attention bid rejected) and 6 uncued (i.e. cue face remained direct after the target bid). Again, these were presented in grayscale, in the centre of the screen, and face dimensions were 219 × 303 pixels.

#### Procedure

##### Phase 1: social value learning

A trial proceeded as follows (see Figure 1D), a fixation cross was presented at the centre of the screen for 1,000 ms, then replaced by the cue and target faces presented on screen together both looking directly ahead (target on left/right counterbalanced) for 750 ms. The target face then looked towards the cue face in a bid for attention (or remained with gaze central in the neutral no-bid condition), presented for 750 ms. The cue face then either looked towards the target face, accepting the bid (high value condition), or looked away from the target face, explicitly rejecting the bid (low value condition) for 500 ms. In the uncued condition, the target face looked towards the cue face, but the cue face remained looking directly. As with the previous tasks, following the attention bid sequence, a question mark appeared above the target face, and participants were required to judge if the target face was older or younger than 25. Participants had 3,000 ms to make the judgement, with the faces remaining on screen for the full 3,000 ms, regardless of when the response was made, to ensure equal exposure time.

##### Phase 2: person evaluation

The person evaluation phase matched that seen in experiments 2 and 3.

### Results

#### Phase 2: person evaluation

Paired samples *t*-tests comparing ratings between the high value (bid accepted) and low value (bid rejected) faces showed no effects of value on any ratings (see Figure 2). Liking (high value, *M* = 5.03; low value, *M* = 5.34), *t*(28) = −1.473, *p* = .152, *d* = −0.274 (*BF*_+0_ = 0.087, i.e. *strong support for H0*); trustworthiness (high value, *M* = 5.16; low value, *M* = 5.56), *t*(28) = −1.835, *p* = .077, *d* = −0.341 (*BF*_+0_ = 0.077, i.e. *strong support for H0*). This therefore indicates that having a bid for attention accepted does not improve the perceived social value of a person compared to having a bid for attention rejected.

## Experiment 5: person evaluation. Target faces have their gaze followed or ignored

In experiment 4, we found that there were no significant effects of seeing attention bids be reciprocated or ignored on ratings of liking or trustworthiness. We did, however, notice that numerically there was a difference in the opposite direction to that predicted, with people giving higher trust ratings to those faces in the low-value social group, that is, those who had their bid rejected. This difference was not significant (*p* = .077) and due to the prediction tested (i.e. that high value faces would be rated more highly than low value faces), the Bayesian analysis results were in favour of the null hypothesis, however, this scenario is interesting to think about in more depth. In experiment 4, the faces that had their bids rejected may have been interpreted as actually having their gaze followed. For example, if the target face is presented on the right, they look to the left to make a bid for attention to the cue, the cue then ignores the bid by looking away and therefore also looking left, thus following the eye gaze of the target face. This scenario could therefore be interpreted as gaze reciprocation with the target being the gaze leader and the cue the gaze follower (Stephenson et al., 2021). This scenario may affect how someone is valued when their gaze is followed. Therefore, in experiment 5, we investigated this directly, by asking whether gaze leading – the act of having one’s gaze followed – increases trust and liking for the lead face. Here the target face always looks away (left or right) from the cue face and the cue face then either follows their gaze (high value condition; gaze leading) or looks in the opposite direction (low value condition; gaze disconnect). We predicted that ratings would be higher for the followed, high-value, faces, compared to the ignored, low-value, faces.

### Method

#### Participants and apparatus

Thirty-eight adult volunteers (8 men, 30 women, mean age 40.9 years, *SD* 14.7 years, range 19–68 years) were recruited from the University of Salford; this was an opportunity sample recruited by a final year project student. Ethical approval was granted by the University of Salford taught ethics board. Participants were recruited via social media and took part online^2^ for no reward. Stimuli were presented using Gorilla (www.Gorilla.sc; Anwyl-Irvine et al., 2020), an online study platform. Participants accessed the study through a web browser using their own desktop/laptop computers.

#### Design

Within-subjects independent variables were social value conditions, (a) High value: gaze is followed. (b) Low value: gaze is looked away from. The experiment had 6 target faces per value signal condition with 8 exposures each (randomised, counterbalanced) = 96 trials (48 trials per value condition). Exposures were reduced here compared to experiment 4 for online study brevity.

#### Stimuli

##### Gaze cue faces

These faces matched the parameters of those used in experiments 2 and 4.

##### Target faces

Here, there were 12 target face identities (6 high value, 6 low value, equal male and female), which matched the parameters of those shown in experiment 4.

#### Procedure

##### Phase 1: social value learning

A trial proceeded as follows (see Figure 1E). A fixation cross was presented at the centre of the screen for 1,000 ms, then replaced by the cue and target faces presented on screen together both looking directly ahead (target on left/right counterbalanced) for 750 ms, then the target face looked away from the cue face for 750 ms. The cue face then either looked towards the target face, thus following their gaze (high value condition), or looked away from the target face, thus explicitly not following their gaze (low value condition). Five hundred milliseconds after this sequence, a question mark appeared above the target face, and participants were required to judge if the target face was older or younger than 25. Due to the online nature of the study, here participants had unlimited time^3^ in which to make the judgement, and the faces disappeared as soon as a response was made.

##### Phase 2: person evaluation

The person evaluation phase matched that used in experiments 2, 3 and 4. Participants also completed the Liebowitz social anxiety scale (Liebowitz, 1987), but we do not analyse this data here^4^ (the data can be found online).

### Results

#### Phase 2: person evaluation

Paired samples *t*-tests showed no significant difference related to social value for trust ratings (high value, *M* = 5.30; low value, *M* = 5.14), *t*(37) = 0.876, *p* = .387, *d* = .142, (*BF*_+0_ = 0.399, i.e. inconclusive evidence) nor for liking ratings (high value, *M* = 5.37; low value, *M* = 5.25), *t*(37) = −0.669, *p* = .508, *d* = .108, (*BF*_+0_ = 0.318, i.e. *moderate support for H0*; see Figure 2). While evidence from trust is inconclusive, it overall shows more support for the null hypothesis than the experimental hypothesis, as does the evidence from the liking ratings. Therefore, this study indicates that having their gaze followed does not improve the perceived social value of a person compared to having their gaze ignored.

### Interim summary

In the previous experiments, we aimed to investigate whether the social context of how someone was initially encountered could affect subsequent judgement of that person through value learning. Across 5 experiments, we found no significant effects of gaze on subsequent liking, trust (or competence, experiment 1) judgements of the faces shown. Importantly, in these experiments, ratings were made in isolation from the gaze cuing sequence. This differs from Bayliss et al. (2006), where judgements were made immediately following the gaze cue (in the final cuing block). We instead aimed to investigate if the effects of engaging in joint attention, or not, when looking at another person are transferred to that person more intrinsically in a longer-lasting fashion, as was seen when investigating willingness to pay for food (Madipakkam et al., 2019). However, it is possible that the effect of joint attention on social value, if it exists at all, is a shorter-lived, temporary effect more akin to that seen for objects in Bayliss et al. (2006). Perhaps early impressions of others are harder to manipulate in the longer term via simple social gaze signals, and for good reason, as this could have very negative consequences if someone can be socially devalued so readily. Therefore, in experiment 6, we used an immediate judgement task for rating the target faces, whereby the ratings were made immediately following the gaze cuing sequence on each trial.

## Experiment 6: immediate judgement task (rate liking immediately after cueing)

This experiment aimed to investigate the effect of joint attention on the immediate social judgement of target faces after they were jointly attended to versus ignored by a cue face. We used an adaptation of the cuing sequence from experiment 2, where the cue face turned their head to look at (high value) or away from (low value) a target face. The target faces always remained looking ahead, and all expressions were neutral. Unlike the previously reported experiments, participants did not judge the age of the target faces, they just rated immediately how much they liked them on a scale of 1 to 9 after they viewed the cuing sequence. Unlike Bayliss et al. (2006) and Landes et al. (2016), here we investigate the effect on liking immediately following the first gaze signal rather than in the final block. We analysed the data as a function of high/low value condition, and as a function of exposure to examine whether liking evaluations changed over time to perhaps indicating the accumulation of social value (each target face was shown and rated 10 times). We predicted that ratings would be higher for the high value compared to the low value faces and that this value effect would build over time, with a larger effect in the final compared to the first block.

### Method

#### Participants and apparatus

We recruited 32 adult volunteers from the University of Aberdeen, however, we removed 1 participant from the sample due to unreliable data, as they used only the numbers 1 and 9 in their ratings in a seemingly random way. Therefore, we had 31 participants in the final sample (4 men, 27 women, mean age 19 years, *SD* 1.86 years, range 17–25 years). All participants had normal or corrected to normal vision, and ethical approval was obtained from the School of Psychology Ethics Committee at the University of Aberdeen. Stimuli presentation equipment matched experiment 1.

#### Stimuli

##### Gaze cue faces

The cue face parameters matched those from experiments 2, 4 and 5, meaning that they made full head turns to look at the target faces.

##### Target faces

Target face parameters matched those in experiment 2; the target faces did not make gaze shifts in this study.

#### Design

Within-subjects’ independent variables were target value, high (always looked at) and low (always looked away from). Again, we also had the uncued condition where the cue face gaze remained direct, but this is not analysed here. The experiment had 6 target faces per value signal condition with 10 exposures each (randomised, counterbalanced) = 180 trials (60 trials per value condition). The dependent variable was the rating of the target faces for liking.

#### Procedure

A trial proceeded as follows (see Figure 1F). A fixation cross was presented at the centre of the screen for 1,000 ms, then replaced by the target and cue faces looking directly for 750 ms, the target face could be on the left or right side, and the cue face was always presented in the centre of the screen. The central cue face was then replaced by the cue face with the gaze shifted left or right, or still showing direct gaze, such that it either looked towards, or away from the target face, or made no eye movement. This was displayed for 500 ms before a question mark appeared above the target face, and participants were asked to rate how much they liked the face now (1 [*not at all*] to 9 [*a lot*]). The faces remained on screen for 4,000 ms with the question mark, such that the faces remained even after participants made their rating. This ensured that faces were shown for the same amount of time in every value condition.

### Results

A repeated measures ANOVA (2 value condition × 10 exposures) showed no significant main effect of value condition (see Figure 2) *F*(1, 30) = 0.574, *p* = .455, η_p_^2^  = .019 (*BF*_inc_ = 0.481, i.e. *anecdotal support for H0*), no significant main effect of exposure (Sphericity violated, Greenhouse-Geisser correction applied) *F*(4.297, 128.924) = 1.704, *p* = .149, η_p_^2^  = .054 (*BF*_inc_ = 0.073, i.e. *strong support for H0*), and no significant interaction between value condition and exposure (Sphericity violated, Greenhouse-Geisser correction applied) *F*(3.825, 114.744) = 0.773, *p* = .540, η_p_^2^  = .025 (*BF*_inc_ < 0.01, i.e. *very strong support for H0*).

### Interim discussion

Across 6 experiments, we have found no evidence that gaze cues can affect explicit person judgement. However, it is possible that gaze cues could affect social value in a more implicit way to influence other forms of social interaction. Therefore, in experiments 7 and 8 we look at implicit effects of gaze on subsequent attention-based interactions. In Phase 1, again, participants made age judgements on identities who were always looked at by gaze cues (high value) and identities that were always looked away from by the cues (low value). Phase 2 now involves an attention orienting task using high/low value faces from Phase 1. In experiment 7, we investigate the effect of gaze cuing on subsequent interactions by investigating how the target face is utilised as a gaze cue. In experiment 8, we investigate the effect of gaze cuing on subsequent interactions by investigating how the target face guides spatial attention in a dot probe task.

## Experiment 7: does being looked at/looked away from affect a person’s ability to guide others’ attention?

Research has found that the gaze cuing effect can be moderated by some social factors, including social status (Dalmaso et al., 2020). Relatedly, work by Capozzi et al. (2016) showed that if in a learning phase an identity was seen to always follow the gaze of others (socially submissive), their gaze was not followed in a subsequent gaze cuing task, whereas for identities that were always followed (socially dominant) there was a gaze cuing effect. Faces were also rated for liking and dominance, but no differences were found for these more explicit ratings. The findings of Capozzi et al (2016), therefore, indicate an implicit social value effect influencing gaze following behaviour potentially related to perceived social power. Therefore, any effects of faces to differentially guide spatial attention according to social value could provide evidence for implicit effects of joint attention on person evaluation.

Here we hypothesised that higher value previously jointly attended faces would subsequently cue attention to a greater degree than lower value previously ignored faces in a gaze cuing task. We conceptualise any value effects built in Phase 1 in terms of ‘cognitive value’ as this is now the nature of the direct measure in Phase 2. However, any influence of high/low value attribution in Phase 1 may also be underpinned by social value perceptions that translate to attention effects.

Because it is unclear if any effects of social power would affect voluntary compared to reflexive processes underlying the gaze cuing effect, we used a non-predictive (50% valid, 50% invalid) and a predictive (75% valid) gaze cuing condition (e.g. Vecera & Rizzo, 2006). This therefore allows us to explore if the effects of value on cuing are only present when the cue is perceived as useful (predictive), or if effects are present when the cue itself has no predictive value. This experiment, including the analysis plan, was pre-registered on AsPredicted: https://aspredicted.org/GW6_TJ6.

### Method

#### Participants and apparatus

We recruited 29 adult volunteers from the University of Aberdeen (opportunity sample in a final year thesis project), however 1 participant failed to respond in Phase 1 so was removed leaving 28 in the final sample (8 men, 20 women, mean age 23 years, *SD* 3.52 years, range 18–32 years). All participants had normal or corrected to normal vision, and ethical approval was obtained from the School of Psychology Ethics Committee at the University of Aberdeen. Stimuli presentation equipment matched experiment 1.

#### Stimuli

##### Gaze cue faces

The cue face parameters matched those from experiments 2, 4 and 5, meaning that they made full head turns to look at the target faces.

##### Targets

In Phase 1, the target face parameters matched those in experiment 2, showing just direct gaze. In Phase 2 where the target faces are used as cue faces, we additionally used the eyes looking left and right natural gaze states. Note these were eye shifts only and not full head turns.

The target in Phase 2 was an asterisk (25 × 25 pixels), presented 110 pixels to the left or right of the cue’s edge.

#### Design and procedure

##### Phase 1: social value learning

Design and procedure matched experiment 2 (see Figure 1B).

##### Phase 2: gaze cuing

Phase 2 within-subjects’ independent variables were predictiveness of gaze cue (predictive: 75% valid; non-predictive: 50% valid), cue value (high value, low value), and cue target validity (valid or invalid). Cue faces with no value (i.e. those from the neutral uncued condition) from Phase 1 were also included but were not analysed. In the non-predictive gaze cuing condition, there were 24 trials per validity condition for each cue face value condition. In the predictive condition, for the valid trials, there were 36 trials per value condition, and for the invalid trials, there were 12 trials per value condition. Predictiveness conditions were presented as separate blocks, that is, all predictive first then non-predictive trials, or vice versa, counterbalanced. At the start of the predictive block, participants were informed using on-screen text that ‘here the cue is informative of the target location 75% of the time’, and at the start of the non-predictive block, they were informed ‘here the cue is NOT informative of the target location’. The dependent variable was median RT to correctly locate the target. To familiarise participants with the task, a 10-trial practice session preceded the main experiment, demonstrating each cue type to the participant, using different faces from those used in the main trials.

**Figure 3.**
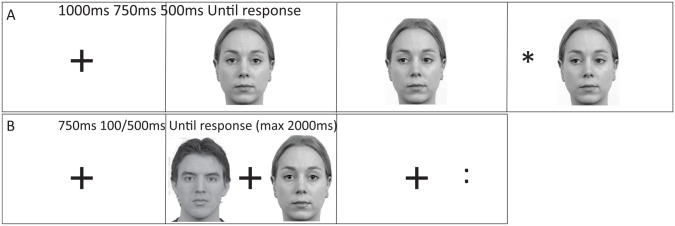
Phase 2: implicit value tasks. *Note.* Figure shows the configuration of the gaze cuing (A) task used in experiment 7 and the dot probe task (B) used in experiment 8 (not to scale). The female face shown is Rafd12, male face shown is Rafd15 from the Radboud database (Langner et al., 2010). Images used in accordance with copyright.

Here the target faces from Phase 1 were used as gaze cues. A trial proceeded as follows. A fixation cross was presented at the centre of the screen for 1,000 ms, then replaced by the direct gaze cue for 750 ms. This was then replaced by the shifted version of the cue (eyes looking left or right; see Figure 3A). After a 500-ms SOA, the target asterisk was presented on either the left or right of the cue until the participant made a response using the keyboard (P if the target was on the right, Q if it was on the left). Participants were asked to respond as quickly and accurately as possible to the target; trials did not time out, and the trial ended when the participant made a response. On valid trials (50% in non-predictive condition, 75% in predictive condition), the target appeared on the side towards which the cue had shifted; on invalid trials (50% in non-predictive condition, 25% in predictive condition), the target appeared on the opposite side. A target was present on all trials. The inter-trial interval was 1,000 ms. Finally, participants completed the EQ (Baron-Cohen & Wheelwright, 2004) questionnaire and the cultural orientation scale (Triandis & Gelfand, 1998), this data is not analysed here but is available online.

### Results

#### Phase 2: gaze cuing

We ran a 2 (predictability) × 2 (value) × 2 (validity) repeated measures ANOVA comparing median RTs across conditions on correct response trials only (96% correct). This showed a significant main effect of cue validity *F*(1, 27) = 11.059, *p* = .003, η_p_^2^  = .291 (*BF*_inc_ = 4.877, i.e. *moderate support for H1*), whereby reaction times were faster when the cue was valid (RTmed = 343 ms) compared to invalid (RTmed = 364 ms). No other main effects or interactions were significant, all *p* values ≥.093, all *BF*_inc_ ≤ 0.328 (i.e. *moderate evidence for H0 and better*), indicating that the value condition (whether they were looked at or ignored in Phase 1) of the face did not influence how people use them as a gaze cue (see Figure 2).

## Experiment 8: does being looked at/looked away from affect a person’s ability to attract others’ attention in an attentional bias task?

The gaze cuing effect may not be an appropriate measure for capturing the implicit effects of social value on future social interaction. While some social factors have been found to influence gaze cueing, such as social dominance, for others, such as trustworthiness, the findings are less clear (Dalmaso et al., 2020). Therefore, here in experiment 8, we use the dot probe task to assess potential implicit effects of social status on subsequent interaction. The dot probe task allows measurement of selective attention in terms of attentional bias. In the task, participants see two lateralised stimuli, one of which is potentially attentionally relevant while the other is less relevant, and then the stimuli disappear from the screen, with a target placed in the location of one of these stimuli. It is predicted that if the participant is biased towards the attentionally relevant stimulus, they will respond more quickly to a target that occurs in the same location as that stimulus (MacLeod et al., 1986). Social effects have been found using this task, for example, people are found to be biased towards happy compared to neutral faces when using a 100-ms presentation time (Wirth & Wentura, 2020). Further, when using the dot probe task to investigate bias towards angry faces in non-clinical populations, effects were found only for rapid, automatic attentional shifts using a 100-ms presentation time, and not for a longer 500 ms presentation time (Cooper & Langton, 2006). Therefore, here we are using the dot probe task to assess if gaze imbues social value influences the attentional importance of the face stimuli, and if this is dependent on presentation time.

Here, Phase 1 was the same as in experiment 7. In Phase 2 target faces from Phase 1 that were always looked at (high value), always looked away from (low value), and neither (uncued) were presented in four pair conditions in a dot probe task: high value – low value; high value – uncued; low value – uncued; uncued – uncued. Only the high value–low value condition was subjected to analyses as this is the core effect of interest. The face pairs were briefly shown on screen followed by two dots that were vertically or horizontally aligned and appeared in the location previously occupied by either the high value, low value or uncued face in each pair. Participants had to state the orientation of the dots as quickly and accurately as possible (vertical/horizontal). Here we hypothesised that we would find an attentional bias preference for the higher value previously attended faces over the low value or uncued faces, reflected in faster correct RTs to identify the dots target when it appeared in the high versus low face locations.

### Method

#### Participants and apparatus

We recruited 49 adult volunteers (paid) from the University of Aberdeen (10 men, 39 women, mean age 23 years, *SD* 3 years, range 18–37 years). All participants had normal or corrected to normal vision, and ethical approval was obtained from the School of Psychology Ethics Committee at the University of Aberdeen. Stimuli presentation equipment matched experiment 1.

#### Stimuli

##### Gaze cue faces

The cue face parameters matched those from experiments 2, 4 and 5, meaning that they made full head turns to look at the target faces.

##### Targets

The target face parameters matched those in experiment 2.

The targets in Phase 2 were 2 dots created using Calibri font size 48 that were either presented in a vertical position (:) or horizontally (. . .)

#### Design and procedure

##### Phase 1: social value learning

Design and procedure matched experiment 2 (see Figure 1B).

##### Phase 2: dot probe task

Here the target faces from Phase 1 were used as paired attention cues. We presented three types of face pairs: (a) high value – low value, (b) high value – uncued, (c) low value – uncued, (d) uncued – uncued (control). The high-low value pairs were the only condition of interest here in line with previous experiments. The target dots appeared equally often in each face location in each pair condition, and the position of the faces within different value face pairs was counterbalanced (i.e. in high value – uncued pairs the high value face was on the left or right side equally and randomly). We also varied the face pair presentation time to either 100 or 500 ms randomly within blocks to measure mechanisms of rapid reflexive orienting (100 ms) and slower, more strategic orienting (500 ms) to the faces (Cooper & Langton, 2006). There were 20 dot probe trials per face pair/SOA condition (240 total trials). An 8-trial practice preceded the main experiment to allow participants to familiarise themselves with the task. This session used a different set of faces to those used in the main study.

A trial proceeded as follows (see Figure 3B). Participants pressed space to initiate each trial, then a fixation cross was shown which stayed on for the full trial. Seven hundred and Fifty milliseconds later, a pair of faces was presented for either 100 or 500 ms. One of these faces was then replaced by a pair of dots, while the other face location was blank. The dots were either horizontally or vertically oriented, and participants were required to press *Z* for horizontal dots and *M* for vertical dots. A trial finished when the participant made a response; however, the trial timed out at 2,000 ms if no response was made. Participants were told that the faces shown were not task relevant, that they should focus on the central fixation cross throughout the trial. Participants’ accuracy and RTs were measured. In addition, participants completed measures of empathy (EQ-60; Baron-Cohen & Wheelwright, 2004) and autistic-like traits (AQ-50; Baron-Cohen et al., 2001); this data is not analysed here but is available online.

### Results

#### Phase 2: dot probe

Here we are primarily interested in the comparison between the high and low value conditions. For the dot probe data, we conducted an ANOVA using only the high- and low-value pair data with presentation time (100, 500 ms) and dot location value (higher of the pair, lower of the pair) as within-subjects variables using median RT data. Incorrect responses were removed (5.6% of data). There was a significant main effect of presentation time, *F*(1, 48) = 15.153, *p* < .001, η_p_^2^  = .240 (*BF*_inc_ = 65.875, i.e. *very strong support for H1*), where RTs to identify dot orientation were faster in the 500 ms than 100 ms condition. However, there were no significant main effect of dot location value (RT Med_high_ = 619 ms; RT Med_low_ = 624 ms; see Figure 2); *F*(1, 48) = 1.229, *p* = .273, η_p_^2^  = .025 (*BF*_inc_ = 0.263, i.e. *moderate support for H0*) nor an interaction between target location value and presentation time; *F*(1, 48) = 0.648, *p* = .425, η_p_^2^  = .013 (*BF*_inc_ = 0.275, i.e. *moderate support for H0*).^5^

### Meta-analysis

Across 8 experiments, we have found no evidence that joint attention, as signalled through gaze cues, positively influences the social or cognitive value of another person. However, it is possible that while the individual experiments show nonsignificant effects, a meaningful trend could be found through mini-meta-analysis. A mini-meta-analysis is simply a meta-analytic method for synthesising the data of a small number of experiments within a manuscript; indeed, it is possible to conduct a meta-analysis with the data from just two experiments. Here we are using the rationale and method as outlined by Goh et al. (2016). Importantly, a min-meta-analysis can help strengthen the evidence that the effect is absent, rather than results being due to a lack of statistical power.

In this article, we have presented 6 studies related to the effect of gaze on explicit liking and trust judgements of others (social value), and 2 studies on implicit attention-based effects (cognitive value). For the mini-meta-analysis, we use the comparison between high and low value status identities only, as this was the focus throughout. For the explicit judgement ratings (experiments 1–5), we look at liking and trust separately, and for the happy versus disgust experiment (3), we use the happy gaze cue condition only (there were no emotion effects). For the immediate judgement experiment (6), where only liking judgments were made, we combined judgements across the 10 exposure time points. For the attention orienting gaze cuing task (experiment 7), we use only the non-predictive cue data, and created difference scores for invalid minus valid RTs so we could compare cuing magnitude for the high- and low-value conditions. Finally, for the dot probe experiment (8), we compare the condition where the high- and low-value faces were paired and combine data for the two presentation times, as there was no interaction here. The prediction throughout was that the higher value faces are perceived more favourably and have greater attentional orienting power.

To conduct the meta-analysis, we used ﬁxed effects in which the mean effect size was weighted by sample size. To do this, we ﬁrst converted Cohen’s *d* into Pearson’s *r* and then Fisher’s *z* transformed these for analyses before converting back to Pearson’s *r* for presentation. For full results, see Table 2. The meta-analysis demonstrates that a high-value face was not liked more than a low-value face, *M r* = −0.023, *Z* = −0.894, *p* = .371, was not trusted more, *M r* = −0.010, *Z* = −0.358, *p* = .720, and was not favoured attentionally, *M r* = 0.088, *Z* = −0.574, *p* = .566.

**Table 2.** Table showing data and results of mini-meta-analysis across the 8 presented studies.

Experiment	*N*	*t*	*df*	*p*	Cohen’s *d*	*r*
Explicit judgement liking						
1 Gaze shift	61	−1.328	60	.189	−0.17	−.085
2 Head turn	25	−0.514	24	.612	−0.103	−.051
3 Happy	30	1.173	29	.25	0.214	.106
4 Bids	29	−1.473	28	.152	−0.274	−.136
5 Gaze following	38	0.669	37	.508	0.108	.054
6 Immediate judgement	31	−0.758	30	.455	−0.136	−.068
Average effect size (weighted)					−0.056	−.028
Combined *Z*						−.894
Explicit judgement trust						
1 Gaze shift	61	−0.515	60	.608	−0.066	−.033
2 Head turn	25	−0.486	24	.631	−0.97	−.048
3 Happy	30	1.326	29	.195	0.242	.12
4 Bids	29	−1.835	28	.077	−0.341	−.168
5 Gaze following	38	0.876	37	.508	0.142	.071
Average effect size (weighted)					−0.022	−.011
Combined *Z*						−.358
Implicit judgement						
7 Gaze cuing	28	−1.121	27	.272	−0.212	−.105
8 Dot probe	49	1.109	48	.773	0.158	.079
Average effect size (weighted)					0.098	.049
Combined *Z*						−.574

*Note*. Values were calculated using the prediction that the higher value faces will be perceived more favourably and have greater attentional orienting power. However, the two tailed test results are presented to show if there are any instances where findings went in the opposite direction to the prediction. Therefore, positive values indicate that the high-value faces were favoured, and negative values indicate the opposite.

## General discussion

The effect of gaze on social judgements and cognitive (attentional value) of others was investigated across 8 experiments. In experiments 1 to 6 we investigated how repeatedly seeing faces either jointly attended (high value condition) or ignored (low value condition) in a Phase 1 gaze cuing task affected subsequent liking and trust judgements of a person. This was conducted both through a value learning approach by delaying judgement to a separate phase (experiment 1–5), and through immediate judgement on each trial (experiment 6). Across these experiments, we found no significant effect of the value context on how the target faces were evaluated, with this being confirmed through meta-analysis. To understand whether value-based effects of gaze on others may occur implicitly, affecting subsequent attentional engagement with others, in experiments 7 and 8, we investigated effects of joint attention on the target faces subsequent ability to exert gaze cuing and attentional bias effects, respectively. For both the gaze cuing task, where high and low value target faces became gaze cues, and the dot probe task, where high- and lowvalue faces served to guide spatial attention, we found no effect of the initial value condition on the degree to which participants followed the gaze of target faces or showed attentional bias. This was also confirmed through meta-analysis.

The findings from experiments 1 to 6 offer a strong evidence base for the argument that while jointly attending to objects can affect how much we may like those items (e.g. Bayliss et al., 2006) joint attention does not appear to affect how we judge other people, or what we call social value. This was found regardless of whether (a) eye gaze shifts or more obvious head turns were used, (b) faces showed happy, disgust, or neutral expressions, (c) target faces bid for initial attention or not (that was then reciprocated or shunned), (d) evaluation judgements were made immediately following the cuing sequence or in a separate phase.

There are several possible reasons why we found no effect of joint attention on person evaluation. As noted by Landes et al. (2016), when they also failed to find an effect of gaze on person judgement, humans inherently elicit affectively valenced reactions in a way that objects do not. Therefore, it is arguably not surprising that judgements of objects can be affected by others’ gaze signals, but judgements of people are more immune. The objects used in the object liking studies (Bayliss et al., 2006) are simple items such as a kettle or a mug, not objects we tend to have strong feelings about. However, effects of joint attention on willingness to pay have been found on food objects (Madipakkam et al., 2019), which are items we may have stronger feelings about in relation to motivational goals such as seeking reward and satiating hunger. When it comes to other people, however, we tend to form rapid first impressions of faces in the absence of other contextual information (Sutherland & Young, 2022; Zebrowitz, 2017). For example, stable inferences about traits such as attractiveness, likeability, trustworthiness, and competence are made after only 100 ms exposure to unfamiliar faces (Willis & Todorov, 2006). Therefore, perhaps these first impressions are impervious to the attention-based signals from others transmitted via gaze to convey social inclusion or exclusion.

It is important to note that the studies presented here deliberately replicate the methods used by Bayliss et al. (2006) in their object liking work. Therefore, the current conclusions may not generalise to other studies investigating the influence of eye gaze on social judgement using alternate methods. Indeed, the findings presented here appear contradictory to those of Kaisler & Leder (2016), who used a naturalistic paradigm and found that individuals looked at by another were rated as more trustworthy than the person looking at them. However, as noted in the introduction, in this study, they did not compare the looked-at individuals to a set of looked-away-from individuals, instead judgements were made against the cue face. Indeed, no gaze effects were found on ratings of trustworthiness or attractiveness in a follow-up study using faces showing emotional expressions where there was a looked-away-from condition (Kaisler et al., 2020). However, they did find a general effect of expression, whereby faces next to an angry face (regardless of expression) were rated as less trustworthy than faces next to a happy face. This indicates that face judgements can be affected by social context, just not eye gaze context.

Our findings also do not align with Jones et al. (2007) who found that male faces looked at by a smiling female face were rated as more attractive than those looked away from by female participants. This demonstrates that gaze may impact preference, however, it can be argued that this does not mean that gaze has affected intrinsic social value. Indeed, in the Jones et al. (2007) study, it can be argued that female participants simply copied the preference of the smiling female cue face, rather than having that cue change their impression of the looked at face. Notably, in this study, faces were compared with ratings for looked-at faces made next to ratings for looked-away-from faces. Further research using comparison measures is required to fully understand these effects of gaze on judgement. It is possible that comparisons require specific higher-level decision-making processes compared to judging an individual alone, which may rely on a faster, more implicit, judgement process (Landes et al., 2016).

Looking to experiments 7 and 8, where we found no effect of gaze cues on the implicit attentional value of the target faces as gaze cues or dot probe cues, it is possible that our behavioural measures used to assess these effects were not sensitive enough. For example, researchers have previously studied the effect of context on attention to faces by pairing faces with neutral or negative information that was either social or non-social in nature, and then used these faces in a subsequent dot probe task (Xu et al., 2016). In that study, it was found that the context of face presentation did not affect the behavioural dot probe result; however, using EEG to measure event-related potentials they found a difference between negative and neutral social contexts, which indicated attentional bias towards the faces in the negative social context. Therefore, future studies should investigate if social value is imbued by gaze cues using neuropsychological methods such as EEG. Of course, it is also plausible that the attempt to imbue target faces with high vs low attentional value via gaze cues in Phase 1 was unsuccessful, perhaps again reflecting the impervious nature of first impressions that also translate to cognitive mechanisms underpinning social interactions.

It is clear from the findings outlined here that relatively simple gaze and head turn cues, even with added emotional expression signals, do not appear to modulate how we perceive and attend to others. Perhaps these signals alone are not sufficient to convey social inclusion or exclusion more fundamentally, so initial first impressions remain regardless of value manipulation. Adding more realistic context in future studies may help to elucidate this further, where there are added benefits to being jointly attended or consequences to being ignored, such as the cyberball task (Williams et al., 2000). In the cyberball task, a target person can be left out of a game, allowing the creation of a more natural social exclusion condition. In the traditional cyberball paradigm, the participant is excluded, often by schematic people without clear and distinguishable identities; however, this can be adapted to exclude an identifiable other, using real faces to enable investigation of the kinds of measures of social and attentional value we employed here.

In Phase 1 of each study, target faces were either looked at (valid cue) or away from (invalid cue) by the cue faces, and we measured the speed with which participants rated the age of the target faces as a function of cue validity. While a gaze cuing effect may be expected, that is, the looked at face being responded to more quickly than the looked-away-from face (Frischen et al., 2007), we found this effect in only studies 2 and 8. This may seem surprising and could be taken to indicate that the reason for the lack of effect on ratings was due to this lack of effect on attention. However, the age task in stage 1 was not a speeded task; indeed, to ensure participants had time to fully engage with the faces in all studies except number 5, the targets stayed onscreen even after participants made a response. In addition, having to think about someone’s age is a more subjective task than the more tangible object categorisation task used by Bayliss et al., (2006), where a RT effect was found. We wanted participants to pay attention to the faces in front of them to make the decision about the age of these individuals. Further, other gaze cuing tasks where effects have been found on higher order processes such as memory have not also included an effect on RT (e.g. Gregory & Jackson, 2017); therefore, the lack of cuing effect on RTs here is unlikely to account for the lack of effect on subsequent person ratings or attention effects.

In conclusion, while previous research has shown some effects of social context upon social value judgements, this does not appear to be directly impacted by eye gaze alone when faces are evaluated in isolation. While the studies presented here do not support our hypothesis that jointly attending to others would result in more positive person evaluation and heighten attention compared to seeing someone being ignored, they do demonstrate a consistent lack of effect across several studies with a variety of manipulations, which is valuable to know. While an individual null result may not be evidence either way of an effect, the consistent lack of an effect seen here is important and shows that while gaze can affect basic object value judgements, it does not affect judgements for faces. This may be a good thing if it protects against unnecessary and potentially harmful manipulation of a person’s social standing through the way others use their eye gaze to include or ignore them.

## Supplemental Material

sj-docx-1-qjp-10.1177_17470218251333425 – Supplemental material for Gaze cues (repeatedly) fail to influence person evaluation Supplemental material, sj-docx-1-qjp-10.1177_17470218251333425 for Gaze cues (repeatedly) fail to influence person evaluation by Samantha E A Gregory, Vilma Pullinen and Margaret C Jackson in Quarterly Journal of Experimental Psychology

## Notes

1. For those interested, analysis using mean RTs is presented in Supplemental Materials (online) for relevant Phase 1 and Phase 2 data analysis across all experiments.

2. Online testing was completed due to ongoing university restrictions related to the COVID-19 pandemic.

3. Note that this resulted in longer RTs in this experiment compared to others for the stage 1 age rating, however, as we are analysing median data, we have not applied any outlier cut offs.

4. Collected to fulfil final year project requirements.

5. Comparable results were also seen for the other conditions (high value compared to uncued and low value compared to uncued), with presentation time (faster in 500 ms condition than 100 ms condition) being the only significant result (*p* < .001, *BF*_inc_ ≥ 24.978). For other comparisons, *p*’s ≥ .573, *BF*_inc_ ≤ 0.182.
