# Supplementary material for: Gaze cues (repeatedly) fail to influence person evaluation
Source: Q J Exp Psychol (Hove). 2025 Mar 28;79(1):124–44. doi: 10.1177/17470218251333425 (PMC12728082; doi:10.1177/17470218251333425)
Supplement: sj-docx-1-qjp-10.1177_17470218251333425 – Supplemental material for Gaze cues (repeatedly) fail to influence person evaluation [file sj-docx-1-qjp-10.1177_17470218251333425.docx]

Supplemental materials: Gaze cues (repeatedly) fail to influence person evaluation.

Authors: Samantha E A Gregory, Vilma Pullinen, Margaret C Jackson

These materials contain:

Table 1. Mean reaction times data for the phase one social value learning tasks

Table 2. Baseline condition social value scores and gaze cuing data for the phase two tasks.

Table 3. Mean reaction times data for the phase two gaze cuing task presented in experiment 7

Table 4. Mean reaction times data for the phase two dot probe task presented in experiment 8, specifically the high and low value pair results.

Note that the mean data analysis was requested as part of peer review and was not part of any planned experimental analysis. All conclusions in the paper are drawn from the median reaction times data. Data using the means generally reflect findings from the median data, however it is important to note that mean data is more susceptible to outlier effects, and no outlier removal criteria were applied here. All data is available on the OSF: <https://osf.io/uzc8p/> , including both the processed and the raw data.

**Table 1**

*Phase 1: Social value learning results using mean data*

|  | High value | Low value | *Uncued* | *t* | *df* | *p* | *d* |
| --- | --- | --- | --- | --- | --- | --- | --- |
| Exp. 1 | 972(135) | 978 (135) | *991 (145)* | 0.671 | 60 | 0.505 | 0.086 |
| Exp. 2 | 889 (221) | 941 (250) | *945 (238)* | 2.183 | 24 | 0.039 | 0.437 |
| Exp. 3 (Happy) | 838 (273) | 850 (279) | - | 0.433 | 29 | 0.668 | 0.079 |
| Exp. 3 (Disgust) | 841 (305) | 867 (296) | - | 0.987 | 29 | 0.332 | 0.180 |
| Exp. 4 | 845 (221) | 846 (224) | *882 (216)* | 0.050 | 28 | 0.961 | 0.009 |
| Exp. 5 | 1964(1074) | 1762 (953) | - | -2.231 | 37 | 0.032 | -0.362 |
| Exp. 7 | 932 (267) | 967 (297) | *1024 (253)* | 1.539 | 27 | 0.135 | 0.291 |
| Exp. 8 | 829 (259) | 863 (292) | *912 (274)* | 2.115 | 48 | 0.040 | 0.302 |

*Note.* Table shows the mean reaction times (standard deviation in brackets) for the looked at, looked away from and uncued conditions, t test results are shown for the comparison between the high value and low value conditions only. For all experiments, the reaction times data was filtered to remove trials where the participant timed out, no other reaction times conditions were applied, data loss due to timing out was < 6% in each experiment. Results reflect those seen in table 1 of the manuscript where median data was sued, except for in experiment 5 where the results shows age ratings were significantly faster in the low value condition. This is likely due to the fact that no outlier removal has been applied, and in the study participants had no response time cut off.

**Table 2**

*Phase 2: Social value scores/ Reaction times (median) data for the uncued baseline condition (SD in brackets)*

|  | Like | Trust | *Comp* |  |
| --- | --- | --- | --- | --- |
| Exp. 1 | 5.51 (1.07) | 5.58 (1.06) | *5.80 (1.10)* |  |
| Exp. 2 | 5.01 (1.30) | 5.17 (1.46) | *-* |  |
| Exp. 3 (Happy) | - | - | - |  |
| Exp. 3 (Disgust) | - | - | - |  |
| Exp. 4 | 5.14 (1.22) | 5.31 (1.16) | *-* |  |
| Exp. 6 (average) | 4.70 (1.18) | - | - |  |
|  | Non-predictive baseline Invalid RT | Non-predictive baseline Valid RT | Predictive baseline Valid RT | Predictive baseline Valid RT |
| Exp 7 | 364 (90) | 331 (57) | 370 (65) | 331 (46) |

**Table 3**

| *Full results for Experiment 7, phase 2 using Mean RTs (comparing high and low value only)* | | | | | | | | | | | | | | | | | | | |  |  |  |  |
| --- | --- | --- | --- | --- | --- | --- | --- | --- | --- | --- | --- | --- | --- | --- | --- | --- | --- | --- | --- | --- | --- | --- | --- |
|  | | | | |  |  | df |  |  | F | | |  | p | | |  | η²_p_ |  | |  |  |  |
| Predictability | | | | |  |  | 1, 27 |  |  | 0.198 | | |  | 0.660 | | |  | 0.007 |  | |  |  |  |
| Value | | | | |  |  | 1, 27 |  |  | 1.441 | | |  | 0.240 | | |  | 0.051 |  | |  |  |  |
| validity | | | | |  |  | 1, 27 |  |  | 24.715 | | |  | < .001 | | |  | 0.478 |  | |  |  |  |
| Predictability ✻ Value | | | | |  |  | 1, 27 |  |  | 0.112 | | |  | 0.741 | | |  | 0.004 |  | |  |  |  |
| Predictability ✻ validity | | | | |  |  | 1, 27 |  |  | 12.426 | | |  | 0.002 | | |  | 0.315 |  | |  |  |  |
| Value ✻ validity | | | | |  |  | 1, 27 |  |  | 1.549 | | |  | 0.224 | | |  | 0.054 |  | |  |  |  |
| Predictability ✻ Value ✻ validity | | | | |  |  | 1, 27 |  |  | 1.006 | | |  | 0.325 | | |  | 0.036 |  | |  |  |  |
|  | | | | | | | | | | | | | | | | | | | |  |  |  |  |
|  | | | | | | | | | | | | | | | | | | | |  |  |  |  |
| **Descriptives** | | | | | | | | | | | | | | | | | | | | | | | |
| **Predictability** | | **Value** | | **Validity** | | | | | | | | **Mean** | | | | **SD** | | | | | | |  |
| Not predictive |  | Low |  | Invalid | | | | | | |  | 385.985 | | |  | 106.079 | | | | | |  |  |
|  |  |  |  | Valid | | | | | | |  | 362.968 | | |  | 77.300 | | | | | |  |  |
|  |  | High |  | Invalid | | | | | | |  | 387.507 | | |  | 139.834 | | | | | |  |  |
|  |  |  |  | Valid | | | | | | |  | 391.541 | | |  | 199.596 | | | | | |  |  |
| Predictive |  | Low |  | Invalid | | | | | | |  | 393.215 | | |  | 92.340 | | | | | |  |  |
|  |  |  |  | Valid | | | | | | |  | 348.615 | | |  | 62.810 | | | | | |  |  |
|  |  | High |  | Invalid | | | | | | |  | 396.152 | | |  | 81.072 | | | | | |  |  |
|  |  |  |  | Valid | | | | | | |  | 363.049 | | |  | 76.731 | | | | | |  |  |
|  | | | | | | | | | | | | | | | | | | | | | | | |

Note that here, in addition to the significant effect of validity seen in the median data, we also see a significant interaction between predictability and validity. This is driven by the predictive condition, whereby the predictive valid condition (356ms) is responded to significantly faster than the predictive invalid condition(395ms; *t*(28) = 6.069, *p_holm_* < .001). Importantly, there is no effect of social value and no interactions.

**Table 4**

*High and low value pair results for Study 8 using mean RT data. Note that results reflect those seen for the median data*

|  | | | | | | | |  | | **df** | | | | | |  | | **F** | | **p** | | **η²_p_** |  |
| --- | --- | --- | --- | --- | --- | --- | --- | --- | --- | --- | --- | --- | --- | --- | --- | --- | --- | --- | --- | --- | --- | --- | --- |
| SOA | | | | | | |  |  |  | 1, 48 | | | | |  |  |  | 17.579 |  | < .001 |  | 0.268 |  |
| target | | | | | | |  |  |  | 1,48 | | | | |  |  |  | 0.207 |  | 0.651 |  | 0.004 |  |
| SOA ✻ target | | | | | | |  |  |  | 1,48 | | | | |  |  |  | 2.242 |  | 0.141 |  | 0.045 |  |
|  | | | | | | | | | | | | | | | | | | | | | | |  |
| *Descriptives* | | | | | | | | | | | | | | | | | | | | | | |  |
| **SOA** | | **target** | | **Mean** | | **SD** | | | | | |  | |  |  |  |  |  |  |  |  |  |  |
| 100 |  | higher |  | 663.319 |  | 99.406 | | | | |  |  |  |  |  |  |  |  |  |  |  |  |  |
|  |  | lower |  | 659.828 |  | 98.960 | | | | |  |  |  |  |  |  |  |  |  |  |  |  |  |
| 500 |  | higher |  | 629.433 |  | 90.384 | | | | |  |  |  |  |  |  |  |  |  |  |  |  |  |
|  |  | lower |  | 637.495 |  | 100.884 | | | | |  |  |  |  |  |  |  |  |  |  |  |  |  |
|  | | | | | | | | | | | | | |  |  |  |  |  |  |  |  |  |  |

Comparable results were also seen for the mean data in the other conditions (high value compared to uncued and low value compared to uncued), with presentation time (faster in 500 ms condition than 100ms condition) being the only significant result (p < .001). For other comparisons, p’s ≥ .485.
